# Partitioned blood pressure polygenic risk reveals differential genetic effects of tissue-specific enhancers and their interactions on cardiovascular disease

**DOI:** 10.21203/rs.3.rs-10406003/v1

**Published:** 2026-08-27

**Authors:** Yihe Yang, Dongwon Lee, Aravinda Chakravarti, Xiaofeng Zhu

**Affiliations:** 1 Department of Population and Quantitative Health Sciences, Case Western Reserve University School of Medicine, Cleveland, OH, 44106, USA; 2 Division of Nephrology, Boston Children’s Hospital, Boston, MA, USA; 3 Department of Pediatrics, Harvard Medical School, Boston, MA, USA; 4 Broad Institute of MIT and Harvard; Cambridge, MA, USA; 5 Center for Human Genetics and Genomics, NYU Grossman School of Medicine, New York, NY, 10016, USA

## Abstract

Polygenic risk scores (PRS) compress genome-wide associations into a single predictor, but this aggregation obscures the distinct biological mechanisms through which genetic variation shapes complex traits. Here we introduce a framework that additively decomposes a trait’s PRS, without loss of SNP heritability, into independent components defined by the tissue-specific and tissue-agnostic cis-regulatory elements (CREs) in which its variants act. Applied to blood pressure (BP) using ~0.5 million CREs across four BP-relevant tissues (adrenal gland, artery, heart, kidney), the framework reveals that regulatory effects are globally additive across tissues yet locally non-additive, and that the resulting partitioned scores carry pronounced, reproducible heterogeneity in their effects on BP and cardiovascular outcomes. We show this heterogeneity reflects gene–environment interactions, and trace one example to its mechanism: a kidney-CRE–partitioned score is protective against coronary artery disease and myocardial infarction through an interaction between *ATP2B1* and antihypertensive medication. Explicitly modeling these interactions improves prediction and transferability, and tissue-focused partitioning increases power to resolve causal genes and reveals genes such as *ADAMTS8* with antagonistic effects across BP components. Validated in an independent *All of Us* cohort, these findings recast the PRS from a blunt aggregate predictor into a mechanistic probe of context-dependent genetic architecture.

## Introduction:

Consider the current enthusiasm for polygenic risk scores (PRS) which use the results of GWAS to provide clinically meaningful prediction of complex diseases.^1–3^ PRSs, calculated as a sum of all trait-influencing alleles weighted by their estimated effect sizes on the trait as provided by GWAS, are attractive owing to their simplicity and considering the genome as one functional unit. However, this simplicity is misleading because the trait-influencing allele effects are tissue dependent ^4^ and likely sensitive to the environment and other factors.^5,6^ PRSs have other challenges to contend with. First, recent studies show that their prediction accuracy decreases substantially when they are applied to individuals whose genetic ancestries are different from those in the training data,^7,8^ limiting transferability across populations owing to differences in linkage disequilibrium (LD), allele frequencies and environmental exposures. Second, although integrating functional annotations can improve PRS predictions,^9,10^ the relevant functions and their interactions are also at the tissue level.^4^ Third, the effect sizes estimated from GWAS are context dependent,^5,6,11^ being affected by gene-environment interactions and thus affect transferability.^5,12^ Despite these recognitions, incorporating the interactions of genetic variants and environments in PRSs is challenging because of low statistical power.^13–15^ This has led to suggestions of conducting pathway-specific PRSs to improve statistical power.^16^

Pathway-level PRS estimation can indeed be useful for partitioning the overall PRS, providing information on which pathways contribute to an *individual’s* susceptibility^17^. For example, it is unknown whether an individual’s risk is largely mono-causal (e.g., renal) or multi-causal (e.g., renal and cardiac), answers with very different implications for disease management. However, systematic evaluation of pathways is also challenging because the term ‘pathway’ is not well-defined,^18^ definitions are not universal and depend on the scientific community involved (e.g., biochemists vs epidemiologists),^19–21^ the number of possible pathways is very large, and pathways have pervasive intercorrelations between them.^18,22–24^ Thus pathway-level PRSs are not additive.

Since the majority of GWAS variants are non-coding and influence the expression of trait-modifying genes by altering transcriptional enhancer activity, often in a tissue- or cell type-specific manner,^25^ it is then reasonable to study the trait contribution of genetic variants by their tissue specific CREs.^4,26^ We hypothesize that variants located in the same type of CREs likely exert their function through similar biological pathways when interacting with environments (contexts). Therefore, partitioning PRSs through tissue-specific CREs offers a systematic, mutually exhaustive and powerful way of studying the origins of disease risk and gene-environment interactions. We refer to this as a *reverse* polygenic risk score (PRS) analysis. Whereas a conventional PRS operates in the forward direction by aggregating trait-associated alleles genome-wide into a single predictive score while discarding the regulatory context of each variant, a reverse PRS operates in the opposite direction: it decomposes that same score into additive, independent components defined by the tissue-specific cis-regulatory elements in which the variants act. In doing so it converts the PRS from a tool for predicting risk into a tool for dissecting the tissue-specific regulation and gene-environment interactions that produce it.

**Fig. 1.**
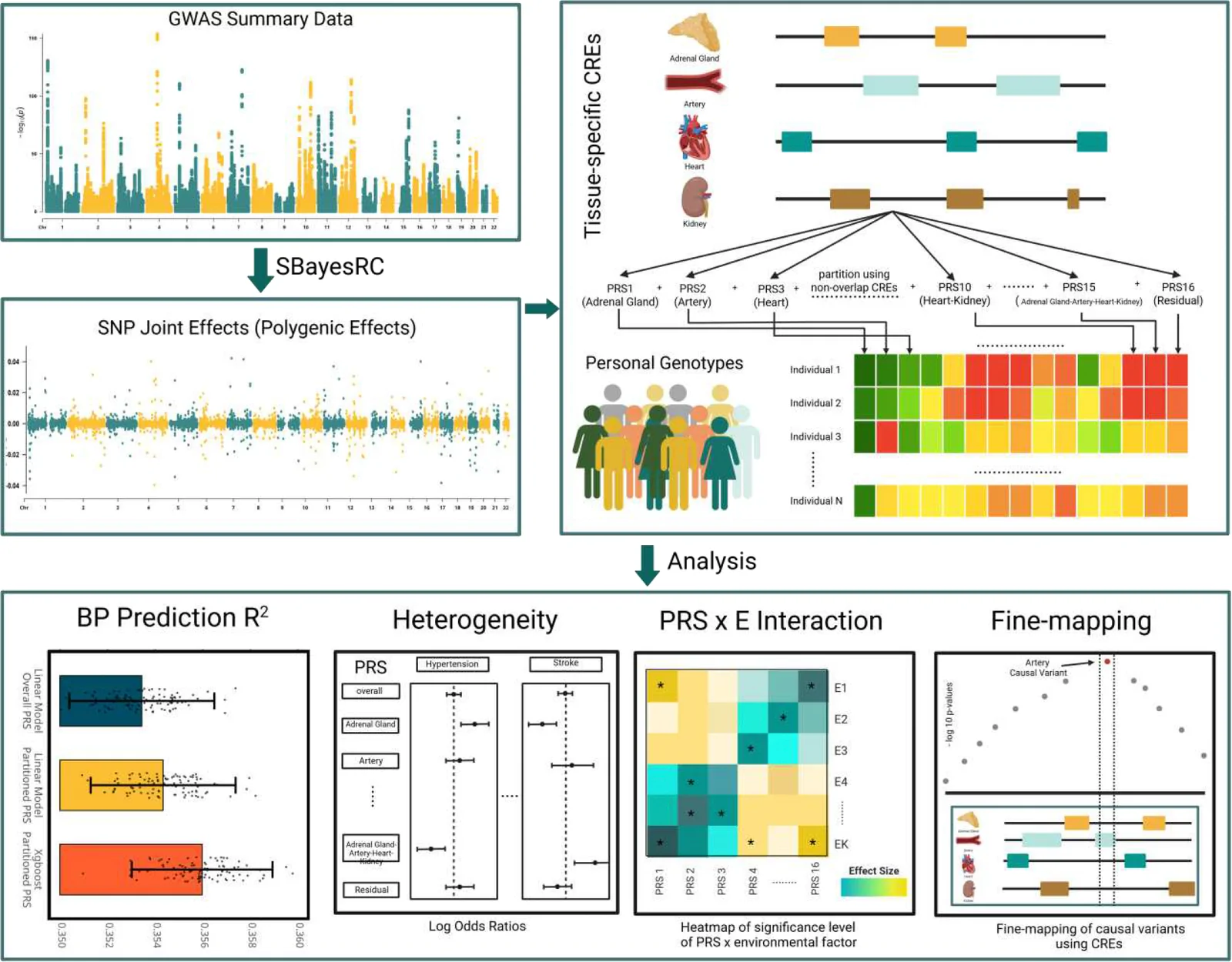
Overview of the analysis pipeline. Software SBayesRC was applied to BP GWAS summary statistics to estimate joint effects of genomic variants after accounting for linkage disequilibrium. The variants were clustered into 16 groups according to the active CREs of the four tissues: the adrenal gland (A), artery (R), heart (H), and kidney (K). BP overall PRS and partitioned tcPRS were calculated using the joint effects of the variants in the UKB data (Methods). The prediction of the overall and tcPRSs were compared for predicting BP traits using linear regression and nonlinear regression (XGBoost). The heterogeneity of the overall PRS and tcPRS was then tested by examining their effect sizes on BP traits and CVD outcomes in UKB. To evaluate whether interactions contribute to the heterogeneity, we further performed interaction analysis between overall and tcPRSs and the 84 variables in the UKB, including demographic factors (age and sex), anthropometric measures (BMI, height), lifestyle factors (sleep, cigarette smoking, alcohol consumption, diet, and physical activity), air pollution, as well as intermediate phenotypes. Replication was performed in AoU. Finally, we investigated whether fine-mapping of BP GWAS loci can be improved by restricting variants in the CREs of the 4 BP tissues.

We partitioned the overall BP PRS by variants located within tissue-specific CREs, here termed tcPRSs. Our overall approach is described in Fig. 1. We selected BP traits because they have been extensively studied in GWAS^27^ and their summary statistics from large cohort studies are publicly available. These partitioned analyses were based on the ~0.5 million human CREs active in four major tissues relevant to BP, namely the adrenal gland (A), artery (R), heart (H), and kidney (K), that we have analyzed and predicted based on our own and publicly available chromatin accessibility data.^4^ Here, we have systematically evaluated their heritability contribution, whether the partitioned PRSs have heterogenous effects in predicting BP and cardiovascular outcomes, and whether they interact with environmental exposures and covariates. Our analyses demonstrate that an individual’s genomic susceptibility can potentially perturb distinct biological pathways and exert heterogeneous effects on target organ damage outcomes. In other words, we may be able to individualize risk at the functional level as well.

## Results:

### Partitioning BP PRSs

**Fig. 2.**
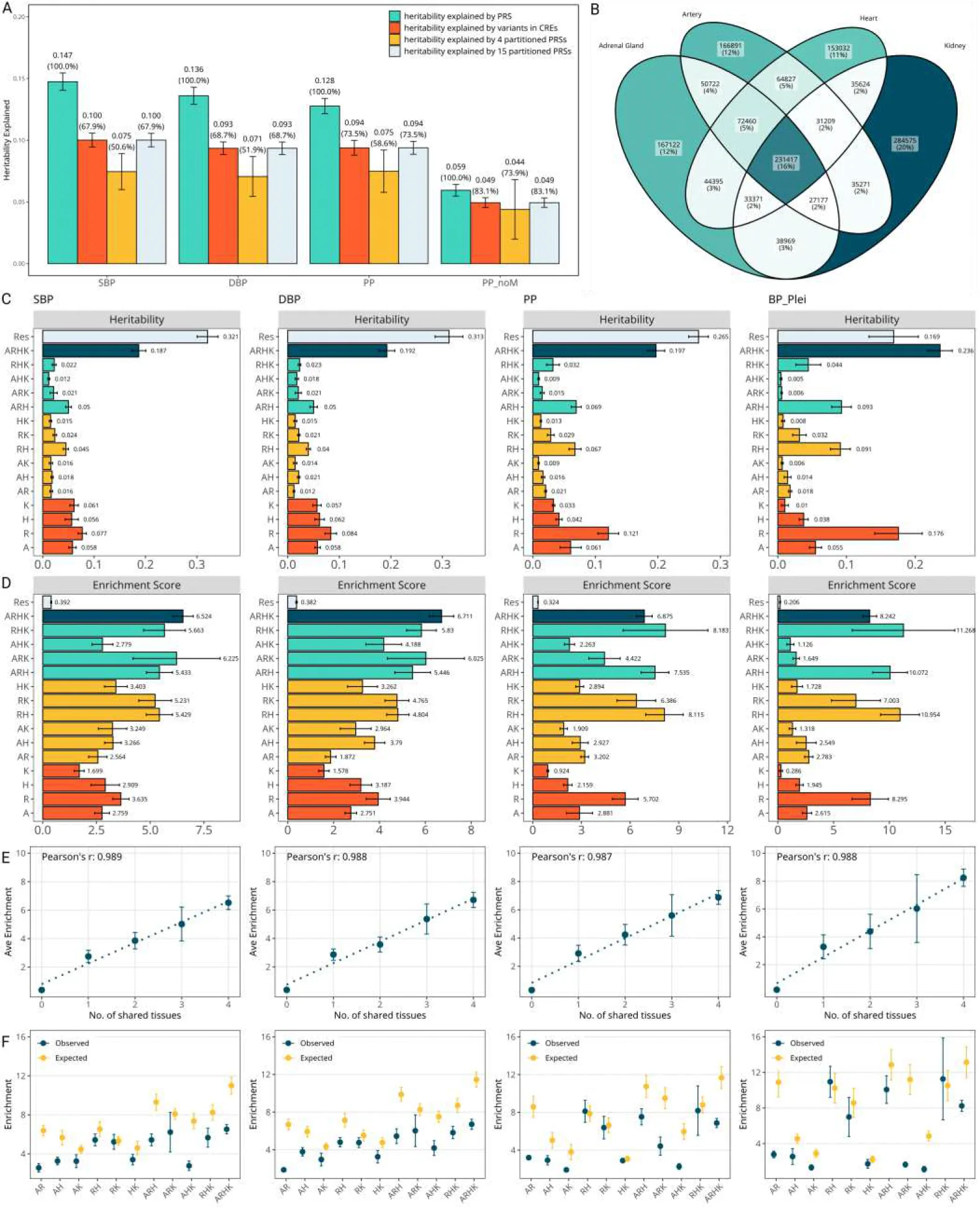
Heritability explained by overall PRS and tcPRSs of SBP, DBP, PP, and BP_plei. **A.** Estimated heritability attributable to the overall PRS (or, SNP heritability), to variants within the union of four tissue-specific CREs, to 4 tissue-CRE partitioned PRS, and to the 15 tcPRSs and resPRS. The heritability of 4 tissue-CRE partitioned PRS was significantly lower than that using variants in the union of four tissue-specific CREs. In comparison, no heritability loss was observed when using the 15 tcPRS. **B**. The distribution of 1,332,197 variants in CREs of the four tissues. **C.** The heritability of the 15 non-overlapping tissue-CRE variant sets and the residual variants for SBP, DBP, PP and PP_plei, respectively. **D.** Corresponding enrichment scores of the 15 non-overlapping tissue-CRE variant sets and the residual variants for SBP, DBP, PP and PP_plei, respectively. **E.** Averaged enrichment scores according to the CREs shared by number of tissues. Each dot represents average enrichment score given the number of shared tissues but ignoring tissue types. **F.** Testing the additivity of enrichment scores by tissue contributions, i.e. (AR=A+R, …ARHK=A+R+H+K). Labels on the Y-axis: A-adrenal, R-artery, H-heart, K-kidney, Res: residual variants.

We first sought to additively partition a BP PRS into multiple components according to the variants located within active CREs of four tissues relevant to BP while preserving heritability. If successful, this framework enables each component to be evaluated independently. A major challenge of such partitioning is that many CREs are tissue-agnostic and shared across tissues. As proof of concept, we partitioned the PRSs of four BP traits, i.e. SBP, DBP, PP, and BP_plei, where BP_plei captures the horizontal pleiotropic effects independent of mediation between SBP and DBP. ^28^ Among the two partitioning strategies evaluated, we found that partitioning based on the 15 disjoint CRE sets defined by these four tissues (Fig. 2 A-B, **Supplementary Note**) preserved both additivity and heritability. We further calculated a residual PRS (resPRS) using all other variants outside the CREs that associated with the four tissues. Accordingly, the overall PRS satisfies $PRS=\sum_{j=1}^{16}tcPRS_{j}$, where j=16 represents the resPRS. Furthermore, the correlations among the 16 tcPRS are low (**Supplementary Fig. 1**), indicating the relative independence between the partitioned genetic components.

As expected, we observed that the resPRS has the largest SNP heritability (20.7%–34.1%), presumably from the 6.02 million variants that map outside the 4-tissue CREs (Fig. 2C). Among the 15 tcPRS, tissue-agnostic CREs accounted for 18.7–23.6% of BP SNP heritability, consistent with our previous study ^4^ and suggesting that substantial BP variation is regulated through variants common to all active CREs of the 4 tissues. CREs specific to the artery account for the second largest BP SNP heritability and the contribution is even more significant for BP_plei (17.6%). The artery active CREs also explain the greatest BP SNP heritability for PP and BP_plei (44.1 and 69.6%, respectively). Heritability is obviously strongly dependent on the number of variants within CREs across the 15 disjoint sets, whereas heritability enrichment is not. For SBP and DBP, heritability enrichment is observed across all 15 disjoint CRE clusters (1.58–6.71 fold enrichment; Fig. 2D). For PP and BP_plei, enrichment is also evident (1.13–11.27 fold), except for CREs active exclusively in kidney tissue, which show no enrichment (0.29–0.92 fold; Fig. 2D), indicating that kidney-specific regulatory effects may be more context-dependent. In contrast, variants outside active CREs are consistently depleted (0.21–0.39 fold), suggesting that BP heritability is largely driven by CREs from these four major tissues. Interestingly, average heritability enrichment scaled almost linearly with the number of BP-relevant tissues in which a CRE is active (Fig. 2E, Pearson’s r = 0.99). However, when we tested whether the enrichment of CREs shared by a specific combination of tissues equals the sum of its single-tissue components (e.g., whether enrichment of AR-CREs = A-CREs + R-CREs), we observed substantial departures from additivity (Fig. 2F). Thus, although BP regulatory influence is additive with respect to tissue count, the effect of any particular tissue combination is non-additive, which is consistent with context-dependent, combination-specific regulatory activity.

### Heterogeneous effects of BP tcPRSs and their interactions

**Fig. 3.**
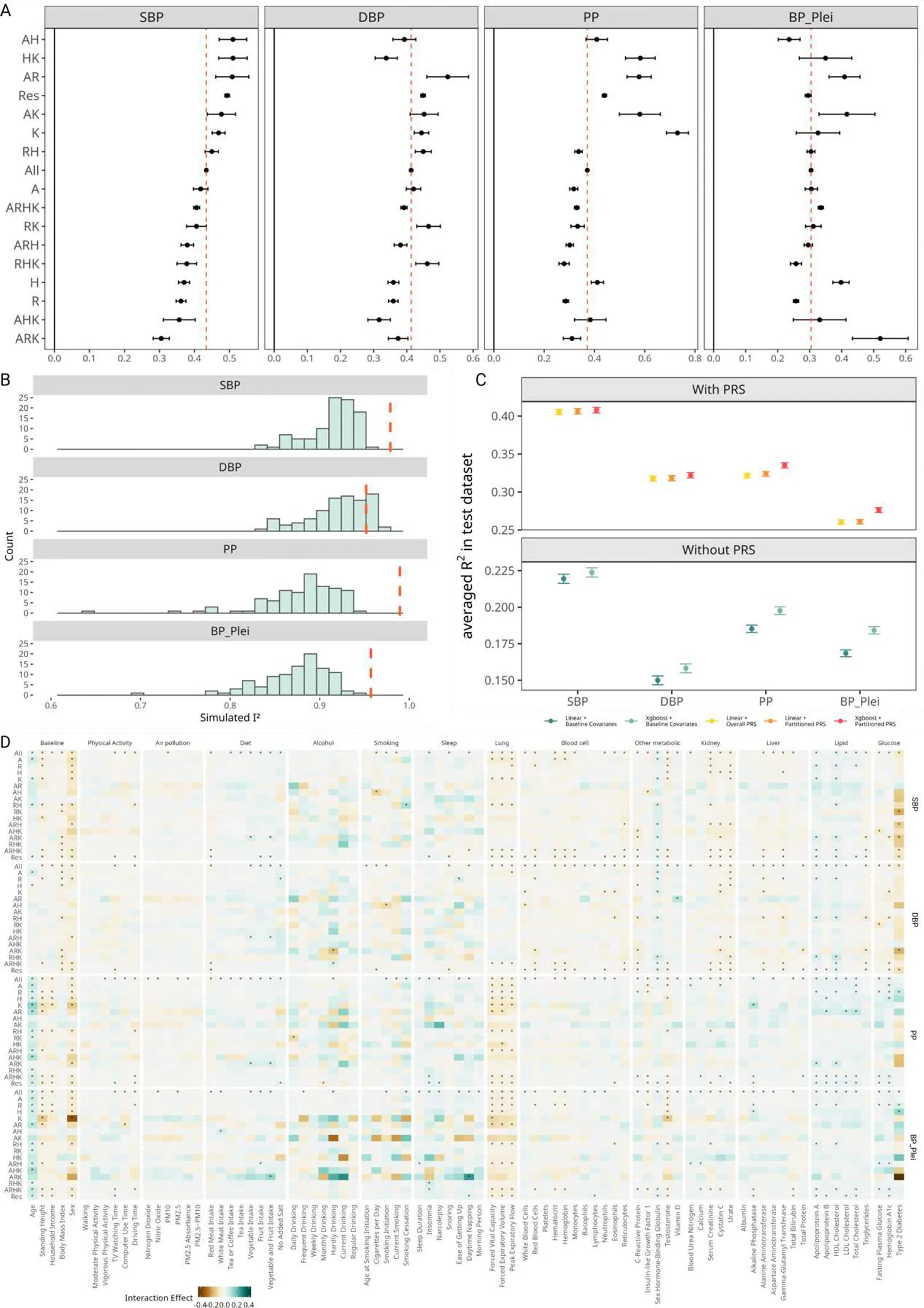
Heterogeneity and interactions of tcPRSs and resPRS on BP traits. **A**: Effect sizes of the 15 tcPRSs, resPRS and the overall PRS (All) on BP traits in UKB. The effect sizes are expected to be the same if there is no heterogeneity. Tissue labels are the same as in Fig. 2 with All representing the overall PRS using all variants. **B**. The distribution of Higgins’ I^2^ by randomly moving the CREs of the four tissues. 100 simulations were generated. At each simulation, tcPRS, resPRS and Higgins’ I^2^ were calculated. The observed Higgins’ I^2^ was marked in red vertical dot line. Except DBP, we did not observe any simulated Higgins’ I^2^ larger than the observed Higgins’ I^2^. **C**: Prediction of overall PRS vs 15 tcPRSs and resPRS for the four BP traits. Top panel: R^2^ of prediction models when including the overall PRS vs including 15 tcPRS and resPRS. Bottom panel: no PRS was included. Points show the average R^2^ across 100 bootstrap samples; error bars denote ±2 standard deviations from 100 simulations. R^2^ was improved by including 15 tcPRSs and resPRS over a single overall PRS, and was further improved by adding nonlinear effects of the tcPRSs and resPRS using XGBoost. **D**: Heatmap of tcPRSs-by-covariate interaction tests. The color scale reflects the magnitude of the interaction effect sizes, and asterisks indicate significant interactions with P < 0.05/16.

Because PRSs have already incorporated the weights for variants, we would expect to observe similar regression coefficients when we perform a linear regression of BP on the overall PRS or on the 15 tcPRS and resPRS simultaneously, as we demonstrated in theory and simulations (**Methods and Supplementary Fig. 2**). Surprisingly, we observed heterogeneity in the estimated regression coefficients of the 15 tcPRSs and resPRS for all the four BP traits, with significant departures from the effect size of the overall PRS in the UKB data (Fig. 3A, **Supplementary Table 2 and 3**). Here, we are less concerned that UKB was included as a subset of the BP GWAS data, since this overlap makes the detection of heterogeneity even more conservative. To assess the magnitude of heterogeneity, we randomly remapped CREs in the genome by keeping their sizes, reconstructed the 15 tcPRSs and resPRS and performed regression analysis in UKB data. We repeated this process 100 times and measured heterogeneity I^2^ scores. The randomly mapped I^2^ scores were never larger than those observed for SBP, PP, or BP_plei, but exceeded observed values in 21 simulations for DBP (Fig. 3B), indicating that the 15 disjoint CRE clusters and the residual cluster capture similar variant functionality for BP traits within clusters but markedly different functionality across clusters although this evidence is less strong for DBP. We also observed significant improved R^2^ values in predicting BP traits using the 15 tcPRSs and the resPRS over the single overall PRS when we applied XGboost^29^ (P<1.17 × 10^−5^), which incorporates nonlinear effects and interactions among the tcPRSs and covariates (Fig. 3C, **Supplementary Table 4**), which suggests the contribution of significant interactions between the tcPRSs and their covariates. Our previous work ^5^ showed that a GWAS marginal effect combines the true main effect with interaction effects. The same holds for a tcPRS. Because different tcPRS components partition variants into distinct regulatory contexts that interact with different environments, their estimated coefficients diverge, producing the heterogeneity we observe even though variant weights were already incorporated.

For clarity of presentation, we added prefixes to the tcPRS to indicate the tissue(s) used in their construction. For example, K-tcPRS denotes a tcPRS constructed from CREs active specifically in kidney tissue, whereas KH-tcPRS denotes a tcPRS constructed from CREs active in both kidney and heart tissues. Analysis of SHapley Additive exPlanations (SHAP)^30^ demonstrated that age consistently emerged as the strongest predictor, followed by the resPRS and the tcPRS constructed through common CREs for all four tissues (ARHK-tcPRS, **Supplementary Fig. 3–6**). Although K-tcPRS explained a relatively lower fraction of SNP heritability, the K-tcPRS of SBP has greater impact than R-tcPRS. Further analysis (**Supplementary Fig. 7**) suggests that this elevated impact is likely driven by a substantial interaction between the K-tcPRS and age, amplifying its contribution in the model.

To further evaluate the contribution of such interactions, we analyzed the relationship between the partitioned tcPRSs and the overall PRS with 84 traits in the UKB, including demographic factors (age and sex), anthropometric measures (BMI, height), lifestyle factors (sleep, cigarette smoking, alcohol consumption, diet, and physical activity), air pollution, as well as intermediate phenotypes. We observed significant interactions between the overall PRS and many of these 84 traits (Fig. 3D, **Supplementary Table 5**). Similarly, we observed substantial numbers of significant interactions between tcPRSs and the 84 traits even after adjusting for the overall PRS (**Supplementary Table 5–7)**. Once again, we observed substantially different interaction effect sizes. The ARHK-tcPRS has most of the significant interactions for all 4 BP traits, i.e., 25 for SBP, 23 for DBP, 20 for PP and 26 for BP_plei among the 84 variables, respectively. Specifically, age shows significant interactions with almost all of the tcPRS for PP and BP_plei except for AH- and AK-tcPRS, which suggests that the effects of the tcPRS vary through age and are consistent with previous studies.^28,31^

It is of note that interactions between sex and the 15 tcPRSs are also widely observed. In particular, K-tcPRS shows strong interactions with sex contributing to SBP, PP and BP_plei but not DBP, which supports the hypothesis that estrogen nuclear hormone effects contribute to sex differences in the genetic architecture of BP,^32^ as well as kidney sodium handling differences between men and women.^33^ We also observed negative height-modified effects of tcPRSs, with stronger modifications for SBP, PP and BP_plei than for DBP. In particular, the K-tcPRS showed the largest negative height-modified effects, which warrants further study. Interestingly, the interaction effects with BMI are only observed for SBP and DBP and not for PP or BP_plei and varies for the tcPRSs. One potential interpretation is that the interaction effects with BMI are similar for SBP and DBP, resulting in the cancellation of their effects when investigating PP and BP_plei since they are both constructed from the difference between SBP and DBP.^28^

In comparison, the interactions with household income are mainly present in PP and BP_plei but not SBP or DBP, highlighting interactions with socioeconomic status (SES). We then examined the interactions of tcPRSs with modifiable environment factors, including physical activity, air pollution, diet, alcohol drinking, smoking and sleep traits (Fig. 3D). Fewer significant interactions with tcPRSs were observed despite significant interactions with overall PRS, which we attribute to low statistical power.

Since many modified environments may also be associated with intermediate phenotypes, we further examined interactions between tcPRSs and BP-relevant intermediate phenotypes, including lung function, blood cell traits, metabolic traits, kidney function, liver function, lipids and glucose traits (**Supplementary Table 1**). We observed a larger number of interactions with intermediate phenotypes than with modifiable environment factors (Fig. 3D). Lung function relevant phenotypes were modified by the genetic effects of tcPRS for SBP, PP and BP_plei the most, while kidney relevant phenotypes were the most modified by PRS effects for SBP and DBP but not for PP or BP_plei, suggesting different pathways are involved for BP traits relevant to lung and kidney functions. We also observed opposite interaction effects between the H-tcPRS of BP_plei and type 2 diabetes (T2D) compared with those of the ARK-tcPRS of BP_plei (0.142 ± 0.047 vs −0.435 ± 0.162). A similar pattern was observed for SBP, DBP, and PP, although the effects were less significant (Fig. 3D). Overall, our findings suggest that interactions with tcPRSs widely exist and their corresponding interaction effects can vary substantially, which contribute to the observed heterogeneity.

### Heterogeneity of BP tcPRS effects on cardiovascular disease (CVD) reveals interaction with antihypertension medication

**Fig. 4.**
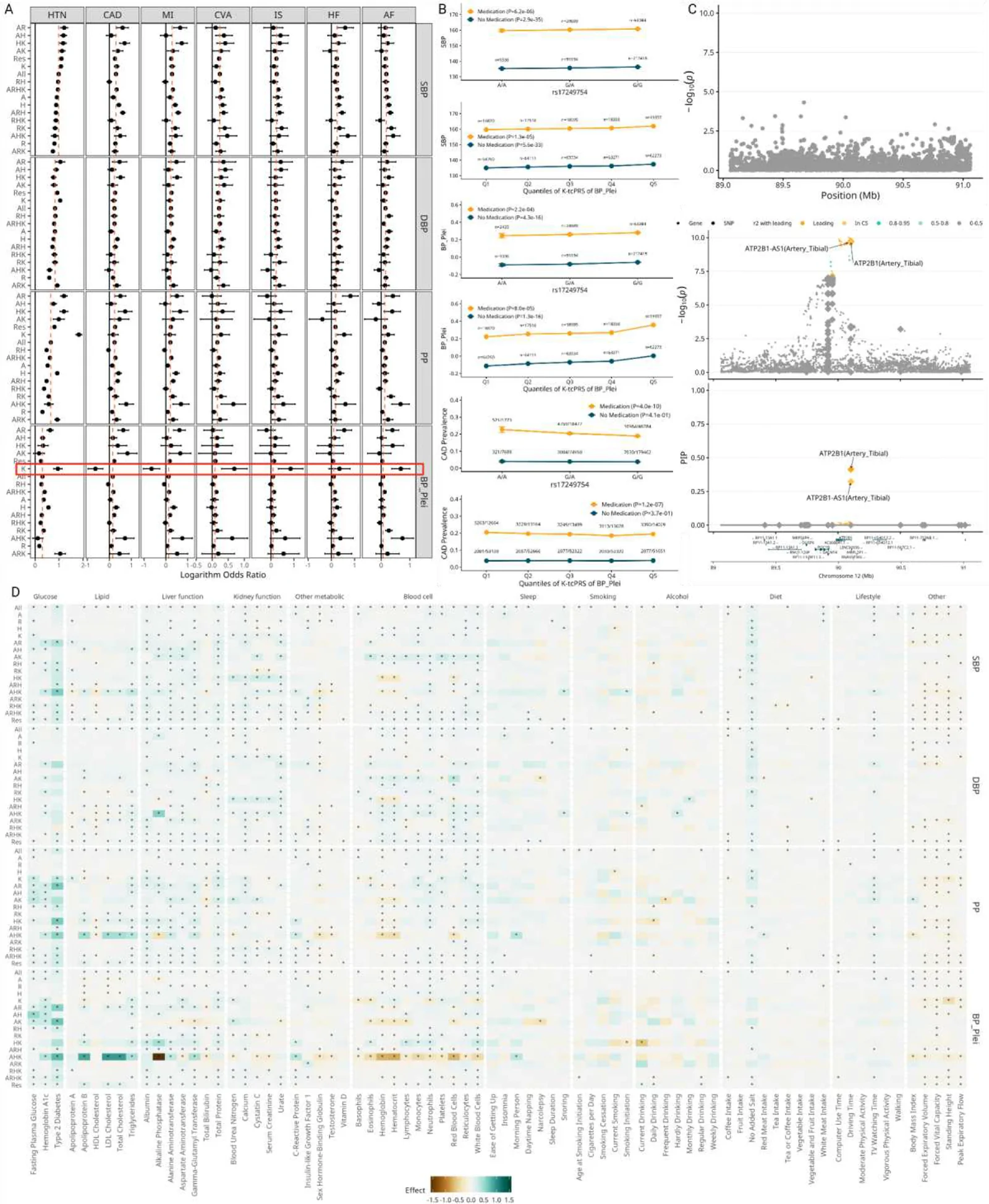
Heterogeneity of tcPRSs and resPRS of BP traits on CVD outcomes and phenome-wide analysis in UKB. **A**. log odd ratios of overall, the 15 tcPRSs and ResPRS on 7 CVD outcomes (HTN: hypertension, CAD: coronary artery disease, MI: myocardial infarction, CVA: cerebrovascular disease, IS: Ischemic Stroke, HF: heart failure, AF: Atrial Fibrillation). The red vertical line indicates the log-odds ratio of the overall PRS, and the black vertical line is the odd ratio of 1. Error bars denote confidence intervals constructed using twice the standard error. The odd ratios of K-tcPRS calculated using kidney CREs and BP_plei were highlighted in red box. Protective effect was observed for both CAD and MI. **B.** UKB Interaction analyses between K-tcPRS and antihypertensive medication on BP traits and CAD, as well as between the intron variant rs17249754 in *ATP2B1* and antihypertensive medication. Analysis was conducted by stratified into medication and no medication group. Interaction was observed on CAD but not on BP traits. For SBP and BP_plei, the numbers represent the sample size. For CAD, the two numbers at each data point represent the CAD cases/controls. All p-values were calculated in linear or logistic regression adjusting for covariates. **C**. Local zoom plot of CAD association at the *ATP2B1* locus by stratified into medication and no medication group. Significant association was only observed in medication group, and fine-mapping using TGVIS was only performed in medication group, resulting that *ATP2B1* and artery tibial are the responsible gene and tissue for the association evidence. **D.** PheWAS associations between the overall PRS, tcPRSs and resPRS with 84 phenotypes. The heatmap color scale reflects the magnitude of the regression coefficients or log-odds ratios, and asterisks indicate associations with P < 0.05/16.

We observed that all four overall BP PRSs significantly increased the risks of related clinical outcomes in UKB (Fig. 4A), prompting us to examine the effects of BP tcPRSs on these same clinical outcomes. Again, we observed substantial heterogeneity in the effects of the tcPRSs across the four BP traits with the general trend indicating increased risk for these clinical outcomes (heterogeneity I^2^ score>28.4%, Fig. 4A, **Supplementary Table 2 and 3**). These effects on outcomes likely reflect associations which may be sensitive to the gene-environment interactions. In contrast, Mendelian randomization estimates are expected to be less sensitive to such interactions. We estimated the effect of each tcPRS on clinical outcomes using only variants located within the CREs defining the corresponding tcPRS. We observed much consistent effects and substantial reductions in heterogeneity I^2^ scores (**Supplementary Fig. 8**), further supporting the contribution of interaction effects to the observed heterogeneity. Notably, the K-tcPRS of BP_plei had an odds ratio 2.59 (95% CI: 2.18–3.08) on HTN, but it had protective effects on coronary artery disease (CAD) (OR: 0.589, 95% CI: 0.424–0.819) and myocardial infarction (MI) (OR: 0.576, 95% CI: 0.398–0.833) (**Supplementary Table 8**). We then screened the variants that lie exclusively within kidney specific CREs and are significantly associated with both BP traits and CAD in previous GWAS studies. We identified an intronic variant, rs17249754 in *ATP2B1*, that lies exclusively within a kidney CRE. The minor allele (G) of rs17249754 was associated with increased SBP, DBP, and BP_plei in previous BP GWAS (effect sizes: 0.0425, P = 1.65 × 10^−144^; 0.0325, P = 2.88 × 10^−68^; 0.0199, P = 1.66 × 10^−26^, respectively)^27^, but was protective against CAD in GWAS (OR = 0.947, P = 1.08 × 10^−17^)^34^. Similar association for this variant with CAD was also observed in the UKB (**Supplementary Table 9**). After conditioning on rs17249754 in UKB, the protective effects of the K-tcPRS of BP_plei on CAD and MI were no longer significant (P > 0.5; **Supplementary Table 10**), indicating that the opposite effects of K-tcPRS on BP traits, HTN, and cardiovascular outcomes are largely attributable to this variant in *ATP2B1*, which has been previously linked to antihypertensive drug response in a mouse model.^35^ Stratified analysis by antihypertensive medication use showed that the protective association of rs17249754 with CAD was present only in the antihypertensive medication group (P=2.9 × 10^−11^), with no association observed in the non-medicated group (p=0.245), and a similar protective pattern was seen for the K-tcPRS of BP_plei (P=2.29× 10^−8^) (Fig. 4B, **Supplementary Table 8–10**), suggesting significant interaction between rs17249754 and antihypertensive medication contribution to CAD. A similar association pattern was observed for MI but not others. Variant rs17249754 is an eQTL of multiple genes including *ATP2B1* in multiple tissues including artery-tibial in GTEx V10^36^. We thus tested whether *ATP2B1* expression in artery tissue at this locus is the most likely gene-tissue responsible for the association signal for CAD and MI in the antihypertensive medication group, using our recently developed fine-mapping method TGVIS^37^. Consequently, we identified *ATP2B1 and ATP2B1-AS1* with artery-tibial in a credible set as the causal gene-tissue (PIP=0.74) that can account for the observed association evidence (Fig. 4C, **Supplementary Table 11**). Similar analysis for myocardial infarction (MI), atrial fibrillation (AF), heart failure (HF), ischemic stroke (IS ) and cerebrovascular accident (CVA) are presented in **Supplementary Fig. 9–13**. Our results suggest that, among patients receiving antihypertensive treatment, carriers of the G allele at rs17249754 or individuals with higher K-tcPRS for BP_plei derive greater cardioprotective benefit, with reduced risks of CAD and MI. Our result is consistent with the observation of less heritability enrichment in kidney specific CREs and context-dependent regulatory effects and further highlights the potential value of partitioned tcPRSs over conventional PRS in uncovering biologically informative pathways and advancing precision medicine for complex diseases.

Finally, we performed PheWAS analysis and assessed heterogeneity of effects of tcPRS across 76 common phenotypes, again revealing widespread heterogeneity (41–53 of these 76 phenotypes were significant with P < 0.05/76 (Fig. 4D, **Supplementary Table 12–13**)).

### Replication in the All of Us (AoU) cohort data

**Fig. 5.**
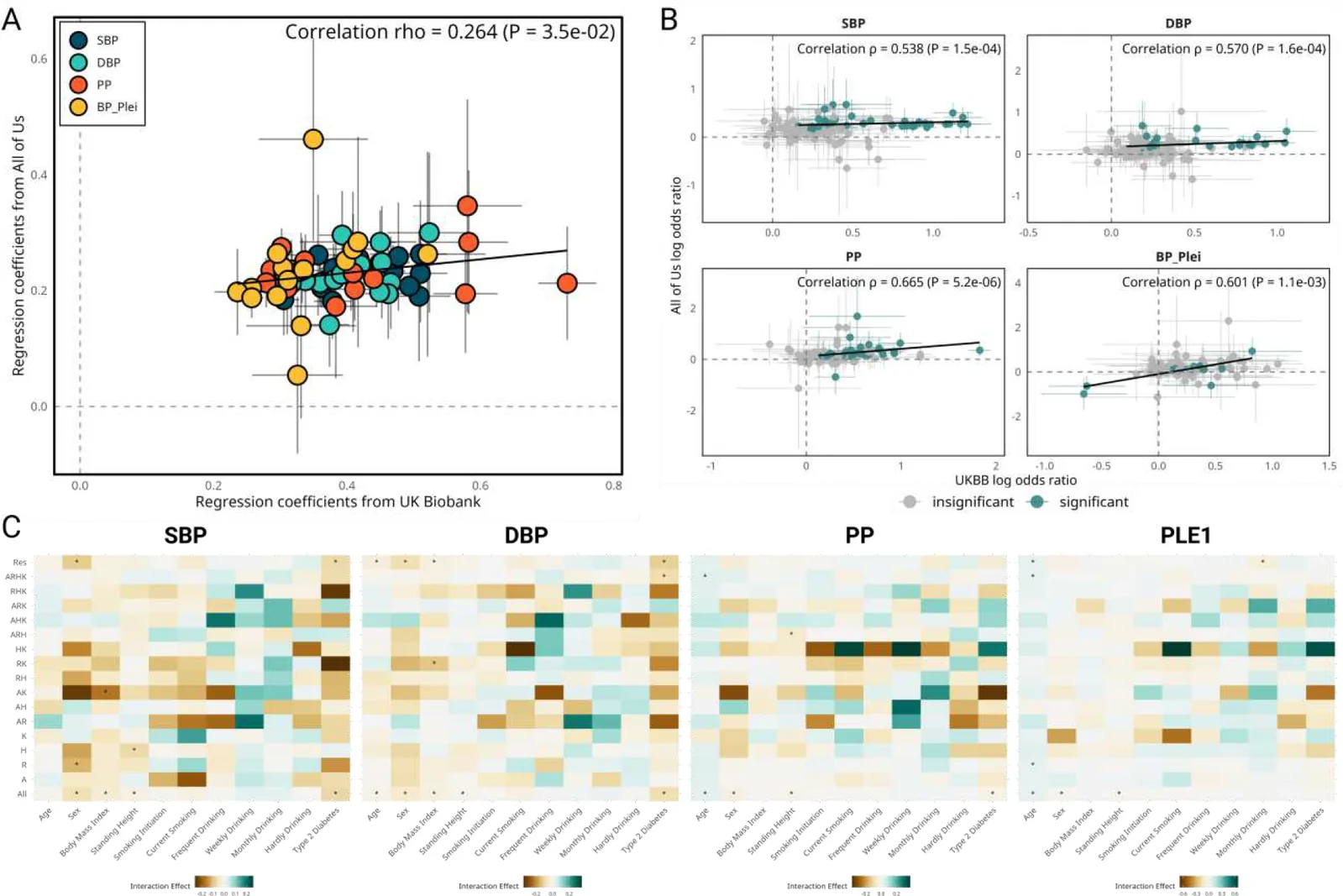
Validation of tcPRS and resPRS associations in the AoU dataset. **A**. comparison of the effect sizes of the 15 tcPRS and the resPRS on BP traits between UKB and AoU. **B**. Comparison of the log-odds ratios of the 15 tcPRS and the resPRS on the 7 CVD outcomes in UKB and AoU. Green Points denote significant partitioned BP PRSs in both datasets, with gray points denote others. Spearman’s ρ is computed using only the significant partitioned BP PRSs. **C**. Heatmap of interactions between the overall and the 15 tcPRS, the resPRS and covariates in AoU. The color scale reflects the magnitude of the interaction effect sizes, and asterisks indicate interaction with P < 0.05/16.

We next sought to examine if we could observe similar heterogeneity of the tcPRSs in European ancestry individuals in the AoU data. We noted that AoU has a smaller European ancestry sample size than the UKB and different distributions of age, sex and environmental factors. As expected, the effects of the tcPRSs despite having larger standard errors still demonstrate significant heterogeneity (**Supplementary Table 14**). We observed significant correlation (0.264, P=0.035) of effect sizes of the tcPRSs on the SBP, DBP and BP_plei between UKB and AoU (Fig. 5A). Restricting this comparison to those significantly associated tcPRSs with clinical outcomes in UKB, the effect sizes were also correlated with those in AoU (0.538 – 0.665), Fig. 5B). Again, we had substantial improved R^2^ values in predicting BP traits using the 15 tcPRSs and the resPRS over the single overall PRS when we used XGboost (**Supplementary Fig. 14**), which is consistent with the observations in the UKB, indicating the existence of significant interactions between the tcPRSs and their covariates. To examine the transferability of PRS in populations other than Europeans, we imputed the European-derived overall PRS, 15 tcPRSs and resPRS into AFR and AMR populations in AoU and evaluated their predictions. Again, we observed significantly improved R^2^ values in predicting BP traits by including partitioned PRSs and interactions using XGboost (**Supplementary Fig. 15 and 16).**

In particular, we again observed significant protective effects of K-tcPRS on CAD (OR:0.637, 95% CI: 0.427–0.951) and MI (OR:0.433, 95% CI: 0.236–0.794), as in the UKB (**Supplementary Table 8**). Importantly, the significance was further improved when combining the evidence from both UKB and AoU. The interaction evidence of K-tcPRS and rs17249754 with antihypertensive medication was further replicated in AoU (**Supplementary Fig. 17**, **Supplementary Table 8–10).** Finally, conditional on the variant rs17249754, the protective evidence of K-tcPRS on CAD and MI was no longer significant (P>0.35). The meta-analysis of UKB and AoU enhanced the association between rs17249754 and CAD (P=1.25×10^−4^) but did not change the significance of K-tcPRS. We observed fewer interactions between tcPRSs and covariates owing to the smaller sample size in the AoU (Fig. 5C, **Supplementary Table 15**), although the patterns were similar.

### Search for causal gene-tissue pairs based on CREs of the four tissues

The above results prompted us to conduct fine mapping of the causal variants within the 4-tissue CREs incorporating gene expression and GWAS results. We searched the causal gene-tissue pairs by joint analysis of GWAS summary data of BP traits^27^ alongside functional quantitative trait loci (xQTL) summary data, including eQTL, sQTL and apaQTL summary statistics from seven GTEx v10 sub-tissues of the adrenal gland, artery, heart, and kidney,^36^ and additionally incorporated our kidney glomerulus and kidney tubule eQTL data from Han et al., ^38^ using the TGVIS software^37^.

We identified 363, 338, 341 and 253 causal gene-tissue pairs for SBP, DBP, PP and BP_plei, respectively, averaging 0.64, 0.62, 0.66, 0.75 pairs per locus (**Supplementary Table 16–19**). The latter proportions are higher than the 0.31 pairs per locus we obtained using 28 tissues,^37^ suggesting that using trait-relevant tissues we can substantially improve the power to detect causal genes. We next examined colocalization evidence for these causal gene-tissue pairs using Coloc-SuSiE.^39^ Across the four traits SBP, DBP, PP and BP_plei, 73.4%−86.1% of the causal gene-tissue pairs showed colocalization evidence (**Supplementary Fig. 18**). Many of these causal genes are also listed as drug-target genes by Trajanoska et al.^40^ (**Supplementary Fig. 19**). In addition to causal gene-tissue pairs, TGVIS identified 70, 82, 74, and 42 direct causal variants for SBP, DBP, PP and BP_plei, respectively, averaging 0.125, 0.152, 0.143, and 0.125 direct causal variants per locus.

**Fig. 6.**
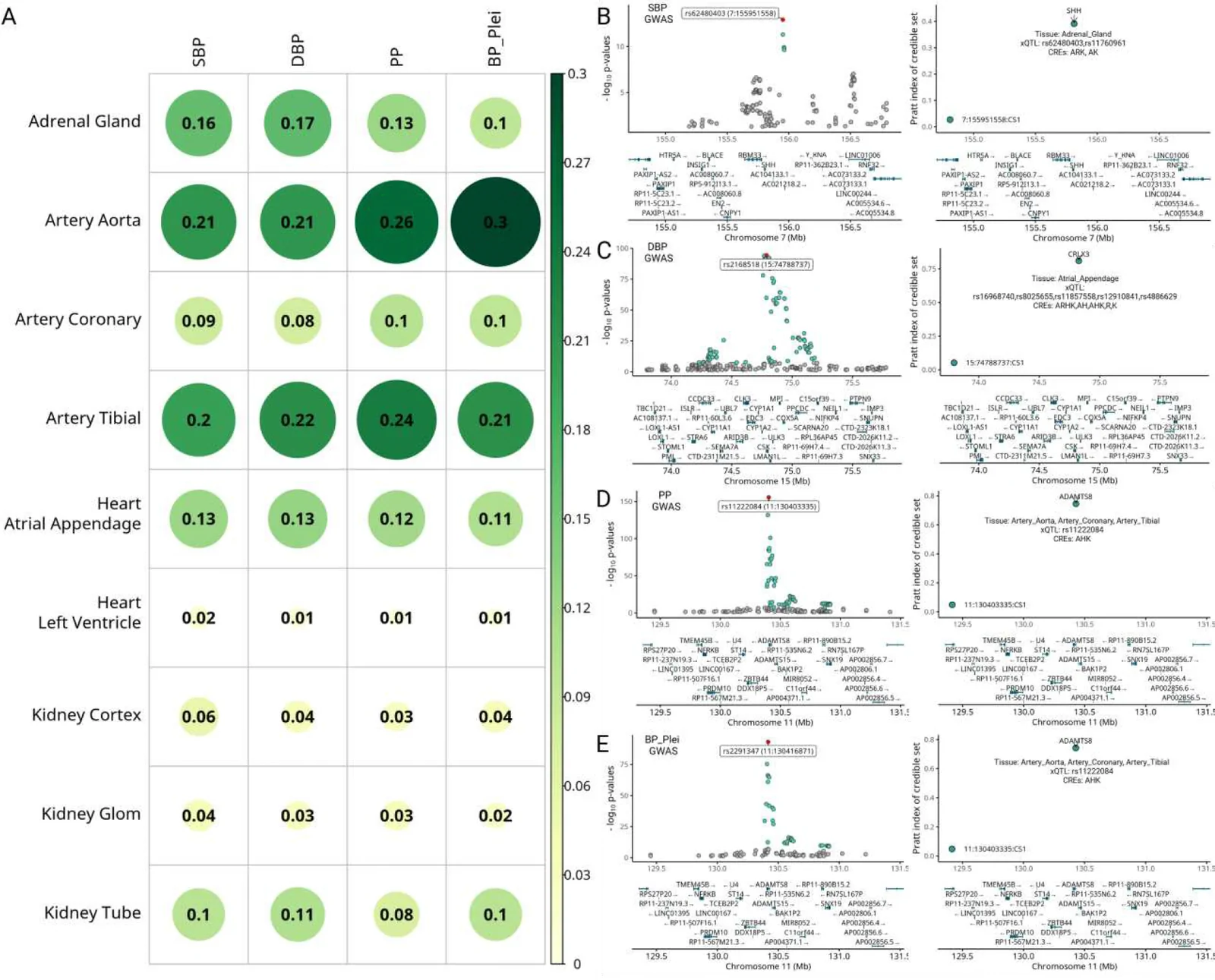
Likely causal gene-tissue pairs and fine-mapping of GWAS BP loci using TGVIS. **A.** Proportion of causal gene-tissue pairs attributable to each tissue for SBP, DBP, PP and PP_Plei. **B-E.** Fine-mapping of four BP loci using TGVIS and four tissue specific CREs. **B**: *SHH* for SBP; **C:** *CPLX3* for DBP; **D:** *ADAMTS8* for PP; **E:** *ADAMTS8* for BP_plei. For each panel, the left sub-panel shows the ’locus zoom’ plot of GWAS results and the right sub-panel presents the fine-mapping results of the gene-tissue pair.

We next estimated the proportion of causal gene-tissue pairs identified by TGVIS for the nine sub-tissue types of the four tissues (Fig. 6A). For all the BP traits, more than 50% causal genes are relevant to artery tissues, and this rate is even higher (>60%) for PP and BP_plei. The remaining three tissues have similar contributions to BP causal genes (10–18%). Atrial appendage and kidney tubule are two dominant subtypes of the heart and kidney tissues for BP genes. Overall, the tissue contributions for BP based on GWAS significant variants are consistent with the partitioned heritability analyses we conducted across the four major BP tissues (Fig. 2).

To examine the biology of the four BP traits, we finally examined the proportions of the xQTLs that lead to identification of causal gene-tissue pairs shared for SBP, DBP, PP and BP_plei (**Methods**). SBP causal gene-tissue pairs were the most widely shared: a large fraction also mapped to DBP, PP, and BP_plei (**Supplementary Fig. 20**). By contrast, DBP pairs were largely DBP-specific (48% shared with neither SBP nor PP; only 7% shared with PP), and PP pairs were similarly PP-specific, regardless of the tissue-partitioned CRE set. BP_plei pairs were even more trait-specific than PP pairs (57% vs. 47%; **Supplementary Fig. 21**). Together, this indicates that SBP biology is broadly shared across BP traits, whereas DBP, PP, and BP_plei each capture a distinct, trait-specific component.

We present four representative examples of causal gene–tissue pairs identified through fine-mapping using TGVIS; the remainder are indicated in **Supplementary Table 16–19**. We identified *SHH* through adrenal gland effects on SBP (Fig. 6B) with its corresponding eQTL residing in an adrenal gland CRE. *SHH* encodes a secreted signaling protein that plays an essential role in embryonic development and tissue homeostasis, particularly in adrenal cortex formation and progenitor differentiation.^41^ Loss of *SHH* function impairs adrenal capsule formation and cortical growth.^42^ Constitutive activation of *SHH* signaling promotes abnormal proliferation of adrenocortical cells and contributes to adrenal hyperplasia leading to primary aldosteronism,^43^ which in turn elevates SBP through aldosterone-driven sodium and water retention and vascular remodeling.^44^

A second novel gene *CPLX3* was identified through its expression in atrial appendage and affects both SBP and DBP (Fig. 6C and **Supplementary Fig. 22**). The association signal corresponds to a splicing variant (chr15:74826867–74828034) within a CRE of *CPLX3* in atrial appendage. However, a second gene (*CSK*) in this region has been nominated as the BP gene.^45,46^ We performed TGVIS again by including subcutaneous adipose tissue because *CSK* is an eGene in adipose tissue. We observed both *CSK*–Adipose Subcutaneous and *CPLX3*–Atrial Appendage as potential causal gene-tissue pairs for SBP and DBP, respectively (**Supplementary Fig. 22**). Follow-up analysis by multi-trait fine-mapping using colocboost^47^ revealed that that *CPLX3*–Atrial Appendage and *CSK*–Adipose Subcutaneous share a colocalization confidence set of coverage 0.95 (**Supplementary Table 20**), suggesting these two genes are statistically indistinguishable but with different tissues.

### *ADAMTS8* exerts antagonistic effects on blood-pressure components

The clearest single-gene illustration of component-specific genetic action is *ADAMTS8*. Across aorta, coronary, and tibial artery, the same CRE-localized eQTL drives its expression (Fig. 6D, 6E), yet its effects on the four BP traits are directionally opposed: it raises DBP while lowering SBP, PP, and BP_plei (**Supplementary Fig. 23**). Such a pattern suggests a redistribution of arterial load characterized by enhanced large-artery compliance (reducing SBP, PP, and BP_plei) and increased peripheral resistance (increasing DBP).^48^ Previous studies of *ADAMTS8* have focused almost exclusively on pulmonary arterial hypertension, where overexpression in pulmonary arterial smooth-muscle cells drives vascular wall thickening and elevated pulmonary pressure.^49^ Our findings extend this paradigm to the systemic arterial system, revealing that *ADAMTS8* exerts bidirectional effects on blood-pressure components rather than a uniform hypertensive action. *ADAMTS8* thus exemplifies why component-level decomposition matters: a gene with antagonistic effects across BP components can appear weak or null in any single composite trait, and becomes interpretable only once the polygenic signal is partitioned.

## Discussion:

In this new PRS analysis framework, we have demonstrated that trait PRS can be usefully additively partitioned into disjoint CRE-based variant sets defined by tissue-specific CRE activity without loss of SNP heritability. Because current BP GWAS has largest sample size in Europeans, we evaluated the heterogeneity of the regression coefficients of tcPRSs on BP traits within European ancestry subjects only, rather than in different ancestries, to reduce the variation that arises from different LD patterns and allele frequencies across ancestries. When there are no gene-environment interactions, we expect no heterogeneity in regression coefficients of the tcPRSs because the variant weights are incorporated in constructing PRSs. However, our results demonstrated significant heterogeneity in regression coefficients of the BP tcPRS (Fig. 3A and 3B) in the UKB. We also showed that such heterogeneity at the tissue-CRE level cannot be explained by different LD and allele frequencies, strongly suggesting the contribution of gene-environment interactions at the tissue-CRE partitioned BP PRS level. This is consistent with the increased R^2^ values we observe when we use XGboost to predict BP levels by including the BP tcPRSs in European populations in UKB and AoU, as well as AFR and AMR populations in AoU, suggesting the improved transferability of tcPRSs. Furthermore, we observe widespread interactions among tcPRSs and covariates, including with sex, age, BMI, environment exposures including alcohol drinking and cigarette smoking, and intermediate phenotypes. In particular, age significantly interacted with almost every BP tcPRS, consistent with the accumulating evidence that PRS performance is context dependent and may be modified by factors such as age, sex, and environment factors.^6,50^ Although genome-wide single-variant scans have found limited evidence for age-modified SBP effects^51^, chromatin accessibility at many CREs is itself age-dependent ^52,53^, so the regulatory impact of variants can shift across the lifespan in ways that aggregate, tissue-partitioned scores are better powered to detect. The age interactions we observe at the tcPRS level are consistent with this, though direct single-cell profiling across ages in adrenal, artery, heart, and kidney would be needed to confirm the mechanism.

We further demonstrated the heterogeneity of BP tissue-CRE partitioned PRS in predicting cardiovascular disease outcomes and a variety of phenotypes (Fig. 4), suggesting that the contribution of BP through BP genes on cardiovascular disease outcomes can be tissue or cell-type specific, consistent with widespread pleiotropy in complex traits.^54–56^ Such heterogeneity and pleiotropy can create additional challenges for estimating the causal effects of risk exposures on disease outcomes in Mendelian Randomization using GWAS without properly addressing these specific gene-environment interactions^5,57,58^. In particular, we observed that the K-tcPRS of BP_plei demonstrated opposite risk effects on HTN and on cardiovascular outcomes, with protective effects on both CAD and MI, reflecting the interaction between K-tcPRS and antihypertensive medication. Higher K-tcPRS of BP_plei was associated with greater cardioprotective benefit among patients receiving treatment, but not among those without treatment. By searching the variants in kidney specific CREs we observed that an eQTL variant rs17249754 in gene *ATP2B1* increased risk of HTN but was protective against cardiovascular outcomes. We were able to localize *ATP2B1* and artery tissue as the potential causal gene-tissue. The opposite risk effect of variants in *ATP2B1* on HTN and CAD was also reported in East Asians.^59^ *ATP2B1* encodes a plasma membrane calcium ATPase 1, and lack of *ATP2B1* gene increases susceptibility to calcium channel blockers than to other types of antihypertensive drugs.^35^ These findings highlight the potential of partitioned PRSs to uncover biologically and clinically meaningful subcohorts, supporting their use in precision medicine.

Our findings indicate that blood pressure heritability is primarily driven by regulatory variation within CREs, with variants outside active CREs showing marked depletion. Notably, CREs shared across multiple BP-relevant tissues exhibit the highest heritability enrichment, highlighting the importance of cross-tissue regulatory programs. In contrast, kidney-specific CREs show limited enrichment, reflecting context-specific effects. Our results also indicate that the overall gene regulatory influences on BP traits can be additive through tissues but understanding the regulatory effects of CREs shared by specific tissues will need to model cell types of tissues. Together, these results support a multi-tissue regulatory architecture underlying BP, dominated by shared CREs with non-additive effects.

Three conceptual multigenic risk models for complex traits and diseases have been recently presented^17^: (1) the polygenic model, which assumes independence between environmental and genetic effects ^60^; (2) the stratagenic model, in which genetic contributions are captured by multiple pathway-specific PRSs and environmental effects are incorporated through different biological pathways ^61,62^; and (3) the omnigenic model, which posits a set of “core genes” that have direct effects on disease and a large set of “peripheral genes” that contribute indirectly by regulating the expression of the core genes.^63,64^ Our framework can serve as the fourth model that differs fundamentally from existing pathway-based approaches. First, we partition a single PRS into tissue-CRE-guided PRSs in an additive and independent manner using genome-wide variants, rather than restricting analyses to genome-wide significant variants from multiple traits, as is common in pathway-specific PRS approaches. Such approaches also require estimating the number of pathways, which is often uncertain and may introduce additional model complexity. Second, our framework provides biological interpretability through gene regulatory networks, as CREs are typically accessed through regulatory interactions that link variants to downstream genes. Third, by substantially reducing the search space, our approach enables more efficient investigation of genetic heterogeneity and gene-environment interactions, as demonstrated in this study. Finally, this framework opens a new avenue for drug development and precision medicine by facilitating the identification of genetically informed treatment interactions, as illustrated by the interaction observed with antihypertensive medication.

One limitation of our study is that we focused on common variants residing within CREs of relevant tissues rather than rare or functional variants within genes. A recent study demonstrated different aspects of trait biology for common and rare variants.^65^ Our study only investigated the tissue-level CREs rather than cell-type level CREs. In addition, our analysis was mainly restricted to populations of European ancestry; extension of this framework to other populations is warranted.

In summary, our study suggests that it is time to rethink the current PRS construction, which treats each variant equally after adding weights obtained from GWAS. It can be more fruitful to cluster variants according to their biological functionality, such as tissue specific CREs, for understanding the disease heterogeneity and identify novel disease pathways, as recently demonstrated in studying the molecular mechanisms of diabetes.^61,62^ Moreover, including gene-environment interactions at partitioned PRS will improve PRS prediction and transferability.

## Materials and Methods:

### UK biobank (UKB):

The UKB individual level data for analyses were accessed through Application ID: 81097. A total of 487,409 individuals with genotype data were initially available. European ancestry individuals were identified using the genetic distance method of Privé et al.^66^. We further randomly removed one individual from each pair with a kinship coefficient greater than 0.0884, resulting in a total of 411,463 participants. Detailed definitions of each disease outcome and the Data Field IDs of all traits analyzed are provided in **Supplementary Table 1**. Due to missing values and other factors, the sample size varies across traits. For SBP and DBP, we used the average of two measurements, either automated or manual, and added 15 mmHg to SBP and 10 mmHg to DBP for individuals on antihypertensive medications. PP was calculated as the difference between the adjusted SBP and DBP values. We then applied an inverse rank normalization transformation (IRNT) to SBP, DBP, and PP to achieve approximately normal trait distributions. For the seven cardiovascular and cerebrovascular diseases considered, controls were defined as UKB participants without these or any other cardiovascular or cerebrovascular conditions. For HTN, T2D, and other binary traits (which were involved in PheWAS analysis), no individuals were excluded.

### All of Us (AoU):

We performed external validation of our results using AoU data, which integrates electronic medical records and participant surveys. We restricted the analysis to genetically inferred European individuals (ancestry probability > 0.95) and excluded related participants (kinship > 0.1), resulting in 178,357 individuals. Physical measurement phenotypes (height, weight, SBP, and DBP) were defined from the earliest visit where all four traits were jointly recorded, retaining only values with correct units and plausible ranges; multiple readings within the visit were summarized by the median, with Office visits prioritized over Outpatient visits. Age was defined as the difference between the measurement date and the participant’s date of birth, sex was defined using sex at birth, and BMI was calculated based on height and weight. In this analysis, we only considered individuals with ages between 40 and 75, which roughly matched the age range of individuals in UKB, and resulted in 98,833 individuals. Use of antihypertensive medications was defined from drug exposure records. Participants were considered users if they had a prescription that either ended before the physical measurement date or overlapped with the same calendar year as the measurement. Smoking status, alcohol consumption, and disease history were defined from participant surveys. Participants were removed if they could not be assigned valid phenotype or covariate information. For genotype data, we calculated variant call rates as the proportion of observed alleles over the maximum possible allele count. Variants with call rates greater than 0.95 were retained. We further compared the MAFs between UKB and AoU and excluded the variants with absolute MAF difference > 0.2. Within the 7.3 million linkage disequilibrium reference panel used in SBayesRC, 6,938,863 SNPs passed this threshold and were included in downstream analyses (**Supplementary Fig. 24**). Phenotypes were standardized following the same procedures as in the UKB. Relevant study details are provided in the **Supplementary Table 1**.

### Summary statistics:

We used the latest GWAS summary statistics on SBP, DBP, and PP, which includes data from over 1 million individuals of European ancestry.^27^ The total sample size for this study was up to 1,028,980 adults, derived from a meta-analysis of four BP-GWAS datasets: the UKB, the International Consortium for Blood Pressure (ICBP), the Million Veteran Program (MVP), and Vanderbilt University’s BioVU biorepository of DNA linked to de-identified medical records. A pleiotropy trait proposed by Zhu and colleagues named BP_plei^28^ was developed to explore the shared as well as distinct genetic components of SBP and DBP. Unlike pulse pressure which is calculated as the difference between SBP and DBP, BP_plei represents pleiotropy contributions after excluding the mediation of one trait on the other. We applied bias-corrected estimating equations (MRBEE)^58^ and estimated the causal effect of DBP on SBP as 0.8 using standardized GWAS summary statistics for SBP and DBP. We then imputed BP_plei as BP_plei = SBP-DBP*0.8.

### Annotation of maps of tissue cis regulatory elements (CREs):

Tissue-specific CRE annotations were generated through reanalysis of DNase-seq and ATAC-seq chromatin accessibility data, using our published methods.^4^ For BP-relevant tissues, including the adrenal gland, artery, heart, and kidney, data were uniformly processed using optimized peak-calling and quality-control with gkmQC, providing higher-quality peaks and stronger heritability signals as compared to publicly available ENCODE HOTSPOT2 peaks. The analyses in this study is based on 7,356,518 common variants studied in Zheng et al.^10^ obtained by matching variants between the UKB, the annotation baseline model BaselineLD v2.2, and the LifeLines cohort. Among these ~7m variants, the number of variants intersecting CREs of the adrenal gland, artery, heart, and kidney are 613,364 (8.34%), 629,972 (8.56%), 616,364 (8.38%), and 661,943 (9.00%), respectively. In addition, variant overlap among the CREs of the four tissues range from 46.2% (artery and kidney) to 59.2% (artery and heart), reflecting varying degrees of shared regulation between these BP-relevant tissues.

### Polygenic risk scores (PRS):

SBayesRC^10^ is a PRS method that integrates functional genomic annotations with high-density variants for polygenic trait prediction. Unlike many other PRS methods that rely on the ~1m variants from HapMap3, SBayesRC utilizes over 7m variants, significantly improving prediction accuracy. Furthermore, SBayesRC refines signals from functional annotations by allowing them to influence both the likelihood of a variant being causal and the distribution of causal effect size. In our analysis, we applied SBayesRC to construct PRSs of SBP, DBP, PP and BP_plei.

### Construction of Tissue-CRE-specific PRS:

We investigated two approaches for PRS partitioning. The first approach constructed 4 tissue-CRE-specific (adrenal gland, artery, heart, and kidney) PRSs, accounting for the CRE overlap among the 4 tissues, using the estimated regression coefficients ${\overset{ˆ}{\theta }}_{1},\;\ldots \;,{\overset{ˆ}{\theta }}_{4}$ in equation (5). We standardized the genetic effect directly estimated by multiplying $\sqrt{2\pi_{j}\left(1-\pi_{j}\right)}$ where $\pi_{j}$ is the allele frequency of the *j*th variant. In the second approach, we partitioned ~0.5 million predicted CREs of the four tissues into 15 disjoint sets based on all possible distinct overlapping patterns among the tissue types in which a CRE is active (Fig. 2B), and constructed PRSs for each category. Variants falling outside of CREs were used to construct the residual PRS.

### Testing heterogeneity of partitioned PRS effects in UKB and AoU:

We performed linear regression on each BP trait using two models:

(1) $$y=\beta_{0}+\beta_{PRS}PRS+\beta_{Age}Age+\beta_{Sex}Sex+\beta_{BMI}BMI+\beta_{HEG}HEG+\beta_{SMK}SMK+\sum_{j}^{15}\beta_{PC_{j}}PC_{j}+\epsilon ,$$

And,

(2) $$y=\beta_{0+}\sum_{j=1}^{16}\beta_{PRS_{j}}PRS_{j}+\beta_{Age}Age+\beta_{Sex}Sex+\beta_{BMI}BMI+\beta_{HEG}HEG+\beta_{SMK}SMK+\sum_{j}^{15}\beta_{PC_{j}}PC_{j}+\epsilon ,$$

Where PRS represents an overall PRS of a BP trait, $PRS_{1}\;\ldots \;PRS_{15}$ represent the 15 tcPRSs and $PRS_{16}$ represents resPRS. Covariates include Age, Sex, BMI, HEG (height), SMK (smoking status) and 15 top ancestry-based principal components PCs. The likelihood ratio test was performed to test no heterogeneity by the hypothesis:

$$H_{0}:\beta_{PRS}=\beta_{PRS_{1}}=\cdots =\beta_{PRS_{16}}.$$

We performed the tests for SBP, DBP, PP, and BP_plei

o separately. For the cardiovascular disease outcomes, we used logistic regression with the same covariates as in equations (1) and (2), and the likelihood ratio test for heterogeneity.

### Permutation-based simulation to assess heterogeneity among tcPRSs

To evaluate whether the observed heterogeneity among tissue-specific PRS components reflects true biological differences rather than random variation, we performed a permutation-based simulation study. For each of 100 iterations, we randomly permuted the assignment of 15 CRE tissue annotations (Adrenal, Artery, Heart, Kidney, and their combinations, plus a residual category) across all variants while preserving the chromosomal positions and SBayesRC-derived effect sizes for four BP traits (SBP, DBP, PP, and BP_plei). For each permuted annotation set, we computed 16 PRS components (15 tissue-specific plus one residual) in the UKB European ancestry cohort (N~350,000 unrelated individuals) using PLINK2. We then regressed each inverse-rank normalized trait on the PRS components, adjusting for age, sex, height, BMI, smoking status, and 15 principal components. Heterogeneity across the 16 components was quantified using the I^2^ statistic from fixed-effect meta-analyses. This simulation generated an empirical null distribution of heterogeneity statistics under the hypothesis of no true tissue-specific effects, against which the observed heterogeneity from the original (non-permuted) annotations could be compared.

### Consistent Regression coefficients when no interactions

For a linear regression model Y=Xβ+ϵ, where Y is the dependent variable vector, X is a matrix of predictors, β is a vector of regression coefficients and ϵ is a random error term, respectively. The least square estimator is $\hat{\beta }={\left(X^{⊤}X\right)}^{-1}X^{⊤}Y$. We now calculate new predictors as $\hat{X}=Xdiag(\hat{\beta })$, where $diag(\hat{\beta })$ is the matrix with the diagonal values $\hat{\beta }$ and 0 for the off-diagonal cells. We now regress Y on $\hat{X}$, resulting in the regression coefficient estimator $\hat{\alpha }={\left({\hat{X}}^{⊤}\hat{X}\right)}^{-1}{\hat{X}}^{⊤}Y=(diag(\hat{\beta }{)}^{-1}{\left(X^{⊤}X\right)}^{-1}X^{⊤}Y=1=(1,\;\ldots \;,1{)}^{⊤}$, suggesting that the regression coefficients the estimated predictors are all equal to constant 1.

When the data to estimate β is different from the data to estimate α, which is the case in our study, we can still perform similar analysis. Let (X,Y) be the data to estimate β, and $\left(\tilde{X},\tilde{Y}\right)$ be the data to estimate α. We have $\hat{\beta }={\left(X^{T}X\right)}^{-1}X^{⊤}Y$ and $\hat{X}=\tilde{X}diag(\hat{\beta })$. In this case, α is estimated by

$$\hat{\alpha }={\left({\hat{X}}^{⊤}\hat{X}\right)}^{-1}{\hat{X}}^{⊤}\tilde{Y}={\left(diag(\hat{\beta }){\tilde{X}}^{⊤}\tilde{X}diag(\hat{\beta })\right)}^{-1}diag(\hat{\beta }){\tilde{X}}^{⊤}\tilde{Y}=diag(\hat{\beta }{)}^{-1}{\left({\tilde{X}}^{⊤}\tilde{X}\right)}^{-1}{\tilde{X}}^{⊤}\tilde{Y}.$$

Clearly, $\overset{ˆ}{\alpha }$ is the ratios of the regression coefficients estimated from data (X,Y) and that from data $\left(\tilde{X},\tilde{Y}\right)$. If (X,Y) and $\left(\tilde{X},\tilde{Y}\right)$ are sampled from the same population, $\overset{ˆ}{\alpha }$ converges to 1. Therefore, we will not expect heterogenous regression coefficients of the predictors unless the regression model is incorrect, such that the interactions among X, and between X and unmeasured covariates in the regression model.

In our study, (X,Y) represent the BP summary statistics and $\left(\tilde{X},\tilde{Y}\right)$ represent the BP UK Biobank or AllofUS data. We used (X,Y) to calculate the regression coefficients of the 15 tcPRS and resPRS. We then performed linear regression between the 16 partitioned PRS and BP traits using data $\left(\tilde{X},\tilde{Y}\right)$. Ideally, we would expect all the regression coefficients $\left(\hat{\alpha }\right)$ of the 16 partitioned PRS as well as the overall PRS are 1. The different BP trait harmonization in different studies such as phenotype transformation can result in $\overset{ˆ}{\alpha }$ being systemically departing from 1. However, all regression coefficients will have a similar scaling, therefore, we should not expect heterogeneity in $\overset{ˆ}{\alpha }$, unless interactions among the 16 partitioned PRSs and their interactions with covariates presented. Here we assume that the BP GWAS summary data have similar linkage disequilibrium patterns (LD) as European ancestry in UK Biobank and AllofUS, and therefore, the impact by different LD is minimal.

### Tests of interactions

We performed the following linear regressions by including interaction terms:

(3) $$y=\beta_{0}+\beta_{PRS}PRS+\beta_{E}E+\beta_{int}PRS\times E+\beta_{Age}Age+\beta_{Sex}Sex+\sum_{j}^{15}\beta_{PC_{j}}PC_{j}+\epsilon ,$$

and,

(4) $$y=\beta_{0}+\sum_{j=1}^{16}(\beta_{PRS_{j}}PRS_{j}+\beta_{int_{j}}PRS_{j}\times E)+\beta_{E}E+\beta_{Age}Age+\beta_{Sex}Sex+\sum_{j}^{15}\beta_{PC_{j}}PC_{j}+\epsilon ,$$

where E refers to environments or intermediate phenotypes in our study. Here y represents the BP traits. **Supplementary Table 1** lists all E factors we analyzed. For continuous E, we applied inverse-rank normalization transformation (IRNT).

### PheWAS analysis:

For continuous phenotypes, we performed linear regression of each phenotype on overall PRS, as well as the 16 partitioned PRSs. We included the covariates of sex, age, and 10 PCs. For binary outcomes, we performed logistic regression using the same covariates.

#### Comparison of linear models and XGBoost for BP prediction

We imputed individual genetic effects using the PRS we constructed in UKB and AoU. Following the approach of Albiñana et al.^67^, we included either the overall PRS or the 16 partitioned PRSs and the same set of covariates: age, sex, BMI, height, smoking status, and the top 15 ancestry-based PCs. We applied XGBoost to fit the models.^29^ We did not perform hyperparameter tuning; instead, we set nrounds = 100, early_stopping_rounds = TRUE, subsample = 0.5, max.depth = 10, and eta = 0.1, keeping all other parameters at their default values. We fitted four models on the training set: (1) a linear model with baseline covariates only, (2) a linear model with baseline covariates and the overall PRS, (3) a linear model with baseline covariates and 16 partitioned PRSs, (4) an XGBoost model with baseline covariates and the overall PRS, and (5) an XGBoost model with baseline covariates and 16 partitioned PRS. The prediction R^2^ of each model was evaluated on the held-out testing set. To compare different models, we implemented a bootstrap procedure. In each iteration, we resampled all individuals with replacement to form the training set and the remaining individuals (known as out-of-bag samples) as the test set. To test the prediction model difference, we applied the corrected resampled t-test of Nadeau and Bengio^68^, which adjusts the variance estimate of the paired R^2^ differences to account for the dependence introduced by overlapping training and testing sets across splits.

### Comparison between UKB and AoU:

We applied the same linear regression for BP traits and logistic regression for cardiovascular and cerebrovascular outcomes, and the interaction models we performed with the UKB data. Based on data availability, we only analyzed age, sex, height, BMI, ever smoking, current smoking, current drinking, regular drinking, and T2D status in AoU. We only included European participants from AoU to reduce confounding. We compared the estimated coefficients between UKB and AoU by calculating Spearman correlations.

### eQTL summary data:

We utilized eQTL and sQTL summary statistics from six tissues provided by GTEx v10, including the artery tibial, artery aorta, artery coronary, heart left ventricle, heart atrial appendage, and adrenal gland.^36^ Additionally, we incorporated eQTL data for tubulointerstitial and kidney glomerular tissues from Han et al.^38^

### Multivariable TWAS analysis using TGVIS:

The Tissue-Gene pair direct causal Variant Infinitesimal effect Selector (TGVIS)^37^ is a multivariable TWAS approach designed to identify causal gene-tissue pairs and direct causal variants, while accounting for infinitesimal effects. TGVIS uses SuSiE for fine mapping these causal elements and employs restricted maximum likelihood to quantify infinitesimal contributions. PIP was used to determine the significance of gene-tissue pairs and direct causal variants, while the Pratt index^69,70^ was used to measure their predictive importance. It should be pointed out, that TGVIS follows SuSiE to group highly correlated or statistically indistinguishable gene-tissue pairs into the same credible set. We treated each credible set as the fundamental unit. For instance, when referencing the number of identified causal gene-tissue pairs, we refer to the number of credible sets.

### Set of variants in fine-mapping of gene-tissue pairs:

For tissue-specific causal gene identification, we limited the analysis to variants located within the four tissue-specific CRE regions identified by Lee et al.,^4^ together with the union of variants reaching P<5×10^−8^ in at least one of the SBP, DBP, or PP GWAS encompassing a total of 1,425,904 variants (Keaton et al.2024).

### Linkage disequilibrium (LD) reference panel:

We identified the least 9,680 unrelated individuals based on kinship coefficients from ~500,000 individuals in the UKBB (Field ID: 22828), comprising 5,205 females and 4,475 males. Genotypes at 1,425,904 variants from these individuals were used to construct a LD reference panel.

### Clumping and thresholding in fine-mapping of gene-tissue pairs:

Our analysis focused on regions within 1mb of genome-wide significant loci for the studied traits. These loci were identified using the clumping and thresholding (C+T) method as implemented in PLINK^71^ by applying additional PLINK parameters: –clump-kb 1000, –clump-p1 5E-8, –clump-p2 5E-8, and –clump-r2 0.01. C+T helps to filter out variants in strong LD, reducing the inclusion of highly correlated or redundant variants, which unnecessarily complicate the models and result in multiple credible sets dominated by these highly associated variants. We assessed the minimum P value of each variant across gene-tissue pairs in eQTL/sQTL data and refined the analysis by applying additional PLINK parameters: –clump-kb 1000, –clump-p1 1E-5, –clump-p2 1E-5, and –clump-r2 0.5. To address the possibility that true causal variants may not be present in the eQTL/sQTL data, we supplemented these variants with outcome GWAS variants meeting P < 5×10^−8^ and r^2^<0.5.

### Removing gene-tissue pairs based on significance in S-PrediXcan:

We used the minimum P value from S-PrediXcan^72,73^ to filter out eGenes or sGenes with P>0.05. This relatively lenient threshold helps to eliminate redundant gene-tissue pairs, thereby reducing the dimensionality of the model. Since our goal was to fine-map causal gene-tissue pairs and identify direct causal variants within GWAS loci with significant signals, this filtering does not introduce a winner’s curse.

### Mapping Major Trait-Relevant Tissues:

For TGVIS^37^, a 95% credible set often contains multiple gene-tissue pairs. To quantify tissue contributions, we calculated the proportion of each tissue present within these sets. This approach allows fractional representation of tissues within a causal credible set of gene-tissue pairs.

### Colocalization of Credible Sets:

We assessed colocalization evidence for the causal credible sets identified by TGVIS. In each region, the same variants used for gene-tissue pair identification were selected for analysis. Fine-mapping was performed on both the outcome and the gene-tissue pairs within the credible sets using SuSiE^74^. We then calculated the posterior probability for hypothesis *H*_4_ (both traits are associated and share a single causal variant) between each outcome and exposure pair using Coloc-SuSiE ^39^. A posterior probability of *H*_4_ > 0.5 was used as the threshold for colocalization evidence between gene-tissue pairs and the outcome. Notably, a single variant meeting this criterion within the credible set was sufficient to establish colocalization.

### Multi-trait Colocalization:

We applied ColocBoost^47^ to perform multi-trait colocalization at the *CPLX3 locus*. We included all common variants in this region, which comprises 3,358 variants. An out-of-sample LD matrix for these variants was computed using European individuals from the UK Biobank. We modeled four traits: SBP, DBP, *CPLX3* expression in Atrial Appendage tissue, and *CSK* expression in adipose subcutaneous tissue. Colocalization was assessed using the default ColocBoost criterion, with a Credible Set of Colocalization (CoS) threshold of 0.95.

## Supplementary Material

Supplementary Files

This is a list of supplementary files associated with this preprint. Click to download.

• SupplemetaryNoteV17.docx

• SupplementaryTableV12.xlsx

• SupplementaryFigureV18.docx

## Data Availability

Blood pressure GWAS summary statistics are publicly available from Keaton et al. (https://www.nature.com/articles/s41588-024-01714-w). Individual-level UK Biobank data are available to approved researchers through the UK Biobank Access Management System(https://www.ukbiobank.ac.uk); analyses in this study were conducted under Application ID 81097. Individual-level All of Us Research Program data are available to registered researchers through the All of Us Researcher Workbench (https://www.researchallofus.org); analyses in this study were conducted on the Researcher Workbench under Controlled Tier access.

## Code availability:

Analysis code will be made publicly available on GitHub and archived on Zenodo with a citable DOI upon publication.
